# Low-dose primaquine to reduce the transmission of *P. falciparum* malaria: a roadmap update

**DOI:** 10.1186/1475-2875-13-S1-P22

**Published:** 2014-09-22

**Authors:** Ingrid Chen, Chris Drakeley, Eugenie Poirot, Jimee Hwang, Roly Gosling

**Affiliations:** 1 Malaria Elimination Initiative, Global Health Group, University of California, San Francisco, San Francisco, CA, USA; 2 Malaria Centre, London School of Hygiene and Tropical Medicine, London, UK; 3 Centers for Disease Control and Prevention, Atlanta, GA, USA

## 

As the only marketed drug that is capable of clearing mature *P. falciparum* gametocytes, primaquine offers a unique ability to stop malaria transmission from humans to mosquitoes. Despite its potential role in malaria control and elimination, the widespread adoption of primaquine has been slow due to its ability to induce hemolysis in G6PD-deficient individuals. While hemolysis is understood to be dose dependent, there are limited data to support the safety and efficacy of current WHO recommendation to give, in conjunction with an artemisinin-based combination therapy, single low-dose primaquine (0.25 mg/kg) to all parasitologically-confirmed *P. falciparum* cases in areas threatened by artemisinin resistance or in settings approaching elimination.

In March 2012, a group of experts from academia, industry, non-governmental organizations, malaria programs, and funders convened to discuss existing data on the use of primaquine as a malaria transmission blocker. The meeting served to identify roadblocks and to propose a roadmap to establish whether low-dose primaquine can be safely and effectively deployed to block *P. falciparum* malaria transmission in sub-Saharan Africa [1].

Since then, much knowledge has been gained on the pharmacokinetics, safety and efficacy of low-dose primaquine. Regulatory barriers to the supply and manufacture of low-dose primaquine in Africa have also been investigated. In March 2014, the group of experts reconvened to discuss roadmap progress and update potential knowledge gaps that need to be addressed in order to determine if and how low-dose primaquine can be implemented as a malaria transmission blocker. We will provide roadmap updates based on the latest meeting and will highlight gaps to welcome discussion and advice from the malaria research community.
